# 靶向CD138嵌合抗原受体修饰T细胞的构建及其抗多发性骨髓瘤作用研究

**DOI:** 10.3760/cma.j.cn121090-20240131-00047

**Published:** 2024-05

**Authors:** 承彩 国, 杨 卢, 克晶 唐, 海燕 邢, 征 田, 青 饶, 敏 王, 冬生 熊, 建祥 王

**Affiliations:** 1 中国医学科学院血液病医院（中国医学科学院血液学研究所）北京协和医学院，血液与健康全国重点实验室，国家血液系统疾病临床医学研究中心，天津市血液病细胞治疗研究重点实验室，细胞生态海河实验室，天津 300020 State Key Laboratory of Experimental Hematology, National Clinical Research Center for Blood Diseases, Tianjin Key Laboratory of Cell Therapy for Blood Diseases, Haihe Laboratory of Cell Ecosystem, Institute of Hematology & Blood Diseases Hospital, Chinese Academy of Medical Sciences & Peking Union Medical College, Tianjin 300020, China; 2 天津医学健康研究院，天津 301600 Tianjin Institutes of Health Science, Tianjin 301600, China

**Keywords:** 嵌合抗原受体, 抗原识别区, 多发性骨髓瘤, CD138, 免疫治疗, Chimeric antigen receptor, Antigen recognition domain, Multiple myeloma, CD138, Immunotherapy

## Abstract

**目的:**

构建一种新型靶向CD138的嵌合抗原受体T（CAR-T）细胞，探索其抗恶性浆细胞肿瘤作用。

**方法:**

通过单克隆抗体制备及筛选技术，获得能稳定分泌CD138抗体的杂交瘤细胞株；将杂交瘤细胞接种至小鼠腹腔，收集腹水并纯化得到抗CD138抗体纯品，进一步检测抗体特异性及亲和力；RT-PCR扩增其可变区序列，以此作为抗原识别域构建CD138 CAR，并表达于T细胞表面，制备CD138 CAR-T；流式细胞术检测CAR-T细胞表型特征；体外杀伤及脱颗粒实验检测其抗肿瘤作用。

**结果:**

①成功制备抗人CD138抗体杂交瘤细胞株，并筛选获得稳定分泌抗人CD138抗体的杂交瘤细胞株5G2。②CD138（5G2）抗体可以特异性识别CD138^+^细胞，与CD138蛋白亲和常数（K_D_）为6.011×10^−9^ mol/L，与CD138^−^细胞无明显交叉反应。③应用分子克隆技术扩增得到CD138（5G2）抗体可变区序列，成功构建CD138（5G2）CAR慢病毒载体，通过感染T细胞获得的CD138（5G2）CAR-T细胞，可以有效结合人CD138重组蛋白。④CD138（5G2）CAR-T可以有效大量扩增，表型检测发现CD138（5G2）CAR-T细胞更多的向中心记忆T及记忆干细胞方向分化，终末分化效应T细胞比例降低。⑤与靶细胞共培养48 h后，与Vector-T细胞相比，CD138（5G2）CAR-T细胞可以有效杀伤CD138^+^骨髓瘤细胞系H929［效靶比为1∶2，（12.92±8.02）％对（54.25±15.79）％，*P*<0.001］。但对CD138^−^ K562细胞系无明显杀伤作用。⑥脱颗粒实验显示，H929细胞可以显著激活CD138（5G2）CAR-T细胞，但对Vector-T细胞无明显激活作用［（25.78±3.35）％对（6.13±1.30）％，*P*<0.001］。⑦与CD138^+^细胞共培养后CD138（5G2）CAR-T分泌细胞因子水平较Vector-T组明显升高［IL-2：（1 697.52±599.05）pg/ml对（5.07±1.17）pg/ml，*P*<0.001；IFN-γ：（3 312.20±486.38）pg/ml对（9.28±1.46）pg/ml，*P*<0.001；TNF-α：（1 837.43±640.49）pg/ml对（8.75±1.65）pg/ml，*P*<0.001］，但与CD138^−^细胞共培养后两组间细胞因子分泌水平无明显差异。

**结论:**

本研究成功制备了抗CD138单克隆抗体，以其抗原识别域构建的CAR-T细胞可以有效发挥抗肿瘤作用，为人CD138抗原的检测及多发性骨髓瘤的免疫治疗提供新的选择。

## Abstract (en)

**Objective:**

To construct a novel chimeric antigen receptor T（CAR-T）cell targeting CD138 and to investigate its cytotoxicity against myeloma cells.

**Methods:**

The hybridoma strain that can stably secrete the CD138 monoclonal antibody（mAb）was prepared and obtained through monoclonal antibody screening technology. The hybridoma strain cells were intraperitoneally injected into mice to produce ascites containing monoclonal antibodies, which were then collected and purified to obtain pure CD138 mAb. Further examinations were performed to assess the biological characteristics of CD138 mAb. The variable region sequence of this antibody was amplified through reverse transcription polymerase chain reaction and was used as the antigen recognition domain of CD138 CAR, which was subsequently expressed on the surface of T cells by lentiviral infection. Flow cytometry was employed to assess the phenotype of CD138 CAR-T cells. In vitro cytotoxicity and degranulation assays were performed to evaluate their antitumor effects.

**Results:**

① We successfully prepared anti-human CD138 antibody hybridoma cell lines and screened a hybridoma cell strain, 5G2, which could persistently and stably secrete the anti-CD138 antibody. ② The purified CD138（5G2）mAb can especially recognize CD138^+^ cells with a binding affinity constant（K_D_）of 6.011×10^−9^ mol/L and showed no significant binding activity with CD138^−^ cells. ③ The variable region sequence of the CD138（5G2）antibody was obtained using molecular cloning technology, and CD138（5G2）CAR was successfully constructed and expressed on T cells through lentivirus infection and, concurrently, demonstrated effective binding to recombinant human CD138 protein. ④ The proliferation of T cells transduced with the CD138（5G2）CAR was highly efficient. The phenotype analysis revealed that CD138（5G2）CAR-T cells exhibited a greater tendency to differentiate into central memory T cells and memory stem T cells, with a reduced proportion of terminally differentiated effector memory subsets. ⑤ CD138（5G2）CAR-T cells demonstrated specific cytotoxicity against CD138^+^ myeloma cell line H929, whereas CD138^−^ cell line K562 remained unaffected. The percentage of residual H929 cells was（12.92±8.02）％ after co-culturing with CD138（5G2）CAR-T cells, while（54.25±15.79）％ was left in the Vector-T group（E∶T＝1∶2; *P*<0.001）. ⑥ Results of degranulation assays demonstrated a significant activation of CD138（5G2）CAR-T cells after co-culture with the H929 cell line, whereas no significant activation was observed in Vector-T cells［（25.78±3.35）％ *vs*（6.13±1.30）％, *P*<0.001］. ⑦ After co-culturing with CD138^+^ cells, CD138（5G2）CAR-T cells exhibited a significant increase in cytokine secretion compared to the Vector-T group［interleukin-2:（1 697.52±599.05）pg/ml *vs*（5.07±1.17）pg/ml, *P*<0.001; interferon-γ:（3 312.20±486.38）pg/ml *vs*（9.28±1.46）pg/ml, *P*<0.001; and tumor necrosis factor-α:（1 837.43±640.49）pg/ml *vs*（8.75±1.65）pg/ml, *P*<0.001］. However, no significant difference was observed in cytokine secretion levels between the two groups after co-culturing with CD138^−^ cells.

**Conclusion:**

This study successfully prepared a novel monoclonal antibody against CD138, and CAR-T cells constructed with the antigen recognition domain derived from this 5G2 mAb demonstrated effective antitumor activity against myeloma cells. This can be used as a new option for the detection of the CD138 antigen and proposes a novel strategy for multiple myeloma immunotherapy.

多发性骨髓瘤（Multiple myeloma, MM）是一种浆细胞恶性肿瘤，其发病率仅次于白血病，是第二常见的血液系统恶性肿瘤，通常表现为骨髓中异常浆细胞大量增殖而引起的骨髓衰竭、肾功能衰竭、高钙血症和骨质破坏[1]。免疫调节药物、蛋白酶体抑制剂以及靶向CD38抗体药物的开发和应用显著提高了MM患者的生存率[2]。然而，对这些药物产生耐药的患者，以及新诊断具有高危遗传学特征或一线治疗后早期复发的患者预后较差，嵌合抗原受体（Chimeric antigen receptor, CAR）T细胞疗法的出现为这部分患者的治疗带来了新的希望[3]。

CAR是包含抗原识别结构域和T细胞信号转导结构域的人工融合蛋白[4]–[5]。通过CAR修饰的患者自身的T细胞，能够识别和攻击特定的肿瘤抗原，以主要组织相容性复合体（Major histocompatibility complex, MHC）非依赖的方式，直接清除靶向的肿瘤细胞[4]–[5]。CAR-T细胞疗法的出现改变了血液系统恶性肿瘤的治疗格局[6]。

CD138（syndecan-1）属于Syndecan家族，在细胞-细胞相互作用、细胞外基质的组装、细胞黏附、信号传导等多种生物学过程中发挥作用，主要表达于细胞表面[7]。CD138过度表达于恶性浆细胞表面，而在正常组织中表达水平较低。更为重要的是，与新诊断病例相比，CD138在复发难治患者中表达水平更高[8]。这使得CD138成为MM治疗的重要靶点。

本研究中，我们成功制备了一种新的抗人CD138单克隆抗体，并以该抗体的可变区序列构建了CD138 CAR，通过体外功能实验初步探讨了CD138 CAR-T细胞在治疗MM中的作用。

## 材料与方法

一、主要材料、试剂与仪器

Balb/c小鼠（8周龄雌鼠）购自北京华阜康生物科技有限公司；DMEM培养基、RPMI 1640培养基、高糖1640培养基、胎牛血清（FBS）、Dynabeads® Human T-Activator CD3/CD28购自美国Gibco公司；Lymphocyte Serum-Free KBM581培养基购自美国Corning公司；重组人IL-2（rhIL-2）购自美国R&D公司；APC抗鼠F（ab）_2_抗体购自美国CST公司；其余所有流式抗体购自美国Biolegend公司；重组人CD138-hFc融合蛋白购于美国Sino Biological公司；NovoCyte流式细胞仪为美国安捷伦公司产品；RNAiso Plus试剂购自日本Takara公司；反转录试剂盒购自北京TransGen Biotech公司；无内毒素质粒抽提试剂盒购自北京TIANGEN公司；限制性核酸内切酶购自美国NEB公司；pMD19-T Simple Vector、T4 DNA Ligase购自日本Takara公司；RosetteSep™人T细胞富集液、HAT半固体筛选培养基购自美国STEMCELL公司；Ficoll样本密度分离液购自天津旷博同生生物技术公司；健康成人外周血标本由天津市血液中心提供。

二、细胞培养

人胚肾细胞系293T培养于含10％ FBS的DMEM培养液中；小鼠骨髓瘤细胞系SP2/0、人浆细胞瘤细胞系H929、人免疫球蛋白A骨髓瘤细胞系MM.1S、人MM细胞系RPMI-8226培养于含10％FBS的高糖RPMI 1640培养基中；慢性髓性白血病细胞系K562、人急性髓系白血病细胞系Molm-13、MV-4-11、Kg1a培养于含10％FBS的RPMI 1640培养基中；人T细胞培养于含10％FBS的KBM581培养基（含50 U/ml rhIL-2）中。

三、抗CD138单克隆抗体的筛选、鉴定及体外活性研究

1. 参照文献[9]中的方法筛选出可以稳定分泌抗CD138抗体的单克隆杂交瘤细胞株5G2。将5G2细胞株接种至Balb/c小鼠腹腔（石蜡预处理），收集腹水经Protein G纯化得到CD138（5G2）抗体纯品，SDS-PAGE凝胶电泳检测抗体纯度，并进一步用于后续抗体亲和力及特异性的检测。

2. CD138（5G2）抗体亲和特异性检测：CD138（5G2）抗体分别与CD138阳性的MM.1S、H929、RPMI-8226细胞系以及CD138阴性的Molm-13、MV-4-11、Kg1a细胞系共孵育后标记APC抗鼠F（ab）_2_二抗，流式细胞术检测其亲和特异性。

3. CD138（5G2）抗体亲和力检测：调整CD138（5G2）抗体纯品终浓度，由200 nmol/L对倍稀释至0.025 nmol/L，分别与2×10^5^ CD138^+^ H929细胞共孵育，后标记APC抗鼠F（ab）_2_二抗，流式细胞术检测其平均荧光强度（MFI），计算CD138（5G2）抗体亲和常数（K_D_）。

四、嵌合抗原受体CD138（5G2）scFv-CD8α-4-1BB-CD3ζ-GFP载体的构建

TRIzol试剂提取5G2杂交瘤的总RNA，逆转录成cDNA文库。通过结合于抗体序列骨架区及信号肽区域的简并引物（表1），RT-PCR法扩增得到抗体序列，连接于pMD19-T载体上。Sanger测序确认CD138（5G2）抗体轻重链的正确序列，并以此为模板SOE-PCR法扩增得到CD138（5G2）scFv片段，克隆至实验室前期构建的pCDH-CD8α-4-1BB-CD3ζ-GFP质粒中[10]–[11]，得到嵌合抗原受体CD138（5G2）scFv-CD8α-4-1BB-CD3ζ-GFP载体。Nhe I和Not I双酶切鉴定成功构建的CD138（5G2）CAR载体。

**表1:** 扩增VL、VH可变区序列简并引物序列

引物名称	引物序列
重链骨架区上游引物	5′-SAGGTGMAGCTKCASSARTCWGG-3′
重链可变区下游引物	5′-TGGGGSTGTYGTTTTGGCTGMRGAGACRGTGA-3′
轻链前导肽上游引物	5′-ATGAAGTTGCCTGTTAGGCTGTTG-3′
轻链可变区下游引物	5′-GGATACAGTTGGTGCAGCATCAGCCCGTTT-3′

五、CD138（5G2）CAR-T细胞的制备

健康供者T细胞的分离纯化：向健康供者外周血中加入RosetteSep人T细胞富集液，以Ficoll密度分离液，离心分离富集CD3^+^ T细胞并检测其纯度。

1. CD138（5G2）CAR病毒及CAR-T细胞制备：无内毒素质粒抽提试剂盒，提取CD138（5G2）CAR及空载Vector质粒。PEI法转染293T细胞系，48 h后收集并浓缩病毒颗粒，感染CD3/CD28磁珠激活24 h后的健康供者T细胞。

2. CD138（5G2）CAR-T及Vector-T感染效率检测：T细胞感染后72 h，取2×10^5^ T细胞与CD138-hFc融合蛋白共孵育，后标记PE-hFc二抗，流式细胞术检测CD138（5G2）CAR-T及Vector-T感染效率。

六、CD138（5G2）CAR-T细胞扩增及表型检测

慢病毒感染T细胞后，每3 d检测CD138（5G2）CAR-T及Vector-T组活细胞绝对计数，分析T细胞增殖情况。

收集感染后96 h CD138（5G2）CAR-T及Vector-T组细胞，分别标记PerCP-Cy5.5抗人CD8抗体、PE抗人CCR7抗体、APC/Cy7抗人CD45RA抗体及相应同型对照抗体，检测两组T细胞的亚型分群情况。

七、CD138（5G2）CAR-T细胞体外功能实验

1. 对MM细胞系细胞毒作用的检测：将CD138（5G2）CAR-T及Vector-T两组细胞分别以2∶1、1∶1、1∶2、1∶4的效靶比与H929（转染RFP）、K562靶细胞共孵育，24 h及48 h后流式细胞术检测其残余靶细胞的比例。

2. 脱颗粒状态检测：将两组T细胞分别以1∶1的效靶比与H929、K562靶细胞共孵育，同时加入50 U/ml rhL-2以及PE/Cy7抗人CD107a抗体。1 h后加入莫能霉素（monensin），4 h后上机检测CD107a^+^ GFP^+^ T细胞比例，反映细胞的激活情况。

3. 细胞因子分泌检测：将两组T细胞分别以1∶1的效靶比与H929、K562靶细胞共孵育48 h后收集共培养上清，免疫荧光法检测上清中IL-2、IFN-γ和TNF-α细胞因子水平。

八、统计学处理

采用GraphPad Prism 9.5.1软件对本实验中所有数据进行计算和统计学分析。所有实验均至少重复3次，数据以均数±标准差进行统计描述，组间差异比较采用two-way ANOVA检验，双侧*P*<0.05为差异有统计学意义。

## 结果

1. CD138（5G2）抗体纯品鉴定及亲和性检测：SDS-PAGE凝胶电泳显示5G2抗体纯品在150×10^3^处呈一单一条带，巯基乙醇还原后可在25×10^3^处见一轻链条带，在50×10^3^见一重链条带（图1A）。CD138（5G2）抗体可以与CD138^+^的MM.1S、H929、RPMI-8226细胞系有效结合，而与CD138^−^的Molm-13、MV-4-11、KG1a细胞系无明显交叉反应（图1B）。该抗体与CD138^+^ H929细胞系的亲和常数为6.011×10^−9^ mol/L（图1C）。

**图1:**
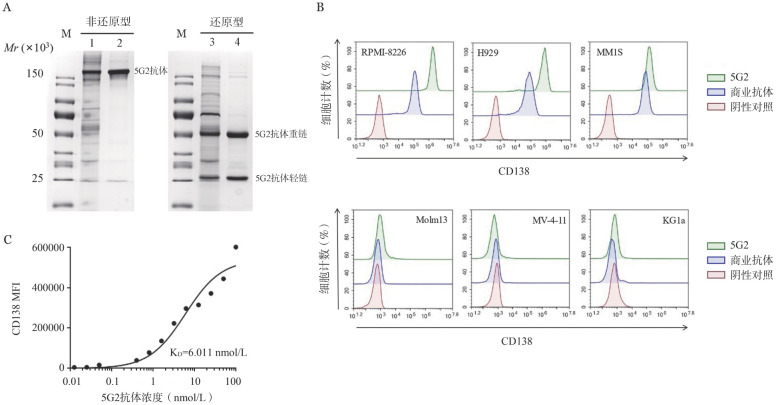
CD138（5G2）抗体纯品鉴定及亲和特性检测 **A** CD138（5G2）抗体纯品SDS-PAGE图。1：5G2粗纯产物；2：5G2纯品；3：5G2粗纯产物；4：5G2纯品；M：Marker。**B** CD138（5G2）单克隆抗体结合特异性检测；**C** CD138（5G2）单克隆抗体亲和常数（K_D_）分析

2. CD138（5G2）scFv-CD8α-4-1BB-CD3ζ CAR表达载体的构建：RT-PCR法从筛选得到5G2杂交瘤中扩增得到CD138（5G2）抗体的轻链及重链序列，通过（G4S）_3_柔性linker将二者相连，成功获得VL-linker-VH方向CD138（5G2）scFv片段，并通过酶切连接到实验室前期构建的pCDH-CD8α-4-1BB-CD3ζ-GFP表达载体中（图2A）。Nhe I和Not I双酶切鉴定可见目的条带（1417 bp）及载体片段（7303 bp）（图2B），且Sanger测序序列比对正确（图2D）。

**图2:**
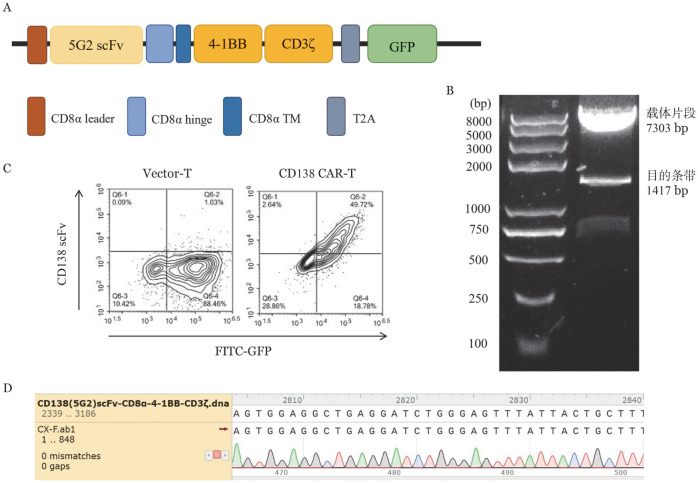
CD138（5G2）嵌合抗原受体（CAR）载体构建 **A** CD138（5G2）scFv-CD8α-4-1BB-CD3ζ CAR载体结构示意图；**B** 载体酶切鉴定琼脂糖凝胶电泳图；**C** 流式细胞术检测Vector-T和CD138 CAR-T细胞感染效率；**D** 载体Sanger测序序列比对图

3. CD138（5G2）CAR-T细胞的制备：慢病毒包装感染正常供者T细胞，GFP荧光检测提示CD138（5G2）CAR及Vector感染效率均大于60％。CD138-hFc融合蛋白标记CD138（5G2）CAR-T及Vector-T细胞，可见CD138（5G2）scFv可以有效结合CD138蛋白（图2C）。

4. CD138（5G2）CAR对T细胞增殖及表型的影响：慢病毒感染T细胞后每3 d检测CD138（5G2）CAR-T及Vector-T组绝对计数，细胞扩增曲线如图3所示，CD138（5G2）CAR-T细胞可以有效大量扩增。

**图3:**
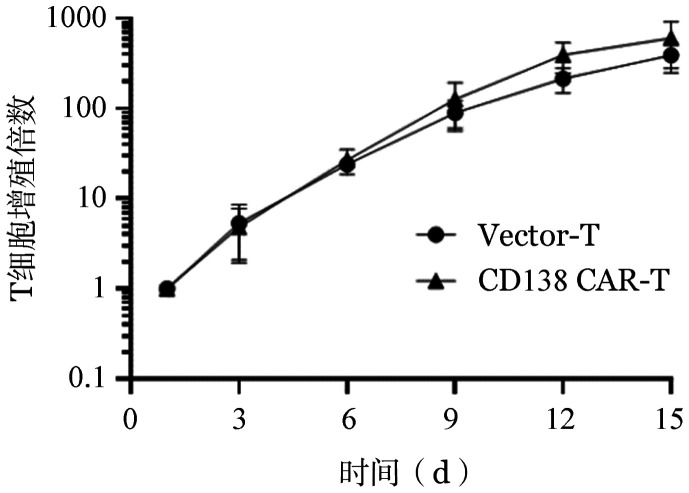
Vector-T和CD138嵌合抗原受体T细胞（CAR-T细胞）增殖情况（实验重复3次）

感染后96 h，流式细胞术检测CD138（5G2）CAR-T及Vector-T组CD4/CD8亚群分布情况如图4A所示，CD138（5G2）CAR-T和Vector-T组CD4^+^ T细胞比例分别为（59.64±2.61）％和（57.47±3.09）％，CD8^+^ T细胞比例分别为（40.37±2.61）％和（42.54±3.09％）％（*P*＝0.486），两组间T细胞CD4/CD8亚群比例差异无统计学意义。

**图4:**
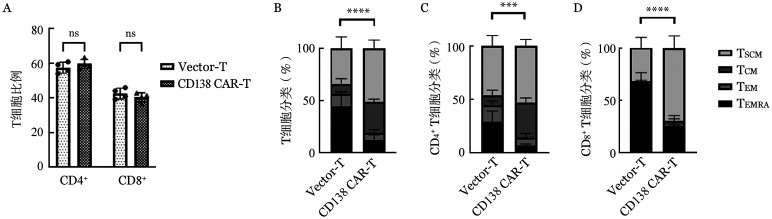
流式细胞术检测Vector-T和CD138嵌合抗原受体T细胞（CAR-T）CD4^+^、CD8^+^ T细胞比例（A）及细胞分化状态（B～D）（实验重复4次，***P<0.001，****P<0.0001，ns示差异无统计学意义） **注** T_SCM_：记忆干细胞T细胞群；T_CM_：中心记忆T细胞群；T_EM_：效应记忆T细胞群；T_EMRA_：终末分化T细胞群

CD45RA及CCR7抗体标记各组T细胞，流式细胞术检测CD138（5G2）CAR对T细胞分化状态的影响（图4B~D），结果显示：与Vector-T组相比，CD138（5G2）CAR-T组终末分化T（T_EMRA_）细胞群比例明显降低（*P*<0.001），而中心记忆T（T_CM_）细胞群和记忆干细胞T（T_SCM_）细胞群比例明显增加（*P*＝0.004、0.016）。在CD4^+^及CD8^+^ T细胞亚群中可见同样的趋势，提示CD138（5G2）CAR-T细胞更多向T_CM_及T_SCM_方向分化。

5. CD138（5G2）CAR-T对CD138^+^骨髓瘤细胞的特异性杀伤作用：流式细胞术检测H929、K562细胞系表面CD138靶抗原表达情况。H929和K562细胞系表面CD138阳性率分别为99.20％和0.53％（图5A），特异性荧光强度（Specific fluorescence intensity, SFI）分别为22.89和0.72（图5B）。

**图5:**
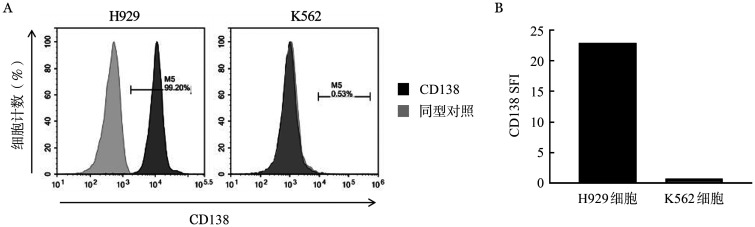
流式细胞术检测肿瘤细胞系CD138阳性细胞比例（A）及特异性荧光强度（SFI）（B）

将H929和K562细胞以2×10^5^/孔接种于24孔板中，分别加入4×10^5^、2×10^5^、1×10^5^、0.5×10^5^浓度（效靶比分别为2∶1、1∶1、1∶2、1∶4）的CD138（5G2）CAR-T及Vector-T细胞，其中H929、K562细胞过表达RFP荧光蛋白。共培养24 h及48 h，流式细胞术检测残余靶细胞比例（图6），结果显示：以效靶比1∶2与阳性靶细胞H929共培养时，24 h后CD138（5G2）CAR-T组残余靶细胞比例为（39.03±7.00）％，明显低于Vector-T组残余细胞比例的（60.35±4.80）％，差异有统计学意义（*P*<0.001）。共培养48 h后CD138（5G2）CAR-T组残余靶细胞比例为（12.92±8.02）％，低于Vector-T组残余细胞比例的（54.25±15.79）％，差异有统计学意义（*P*<0.001）。而与阴性靶细胞K562共培养后，CD138（5G2）CAR-T组与Vector-T组残余细胞比例差异无统计学意义，共培养48 h时2∶1、1∶1、1∶2、1∶4效靶比残余靶细胞比例分别为（54.63±13.16）％对（57.43±9.59）％（*P*＝0.974）、（71.99±8.74）％对（76.21±8.57）％（*P*＝0.893）、（87.41±3.34）％对（88.13±2.86）％（*P*＝1.000）、（93.80±1.28）％对（93.45±1.91）％（*P*＝1.000）。

**图6:**
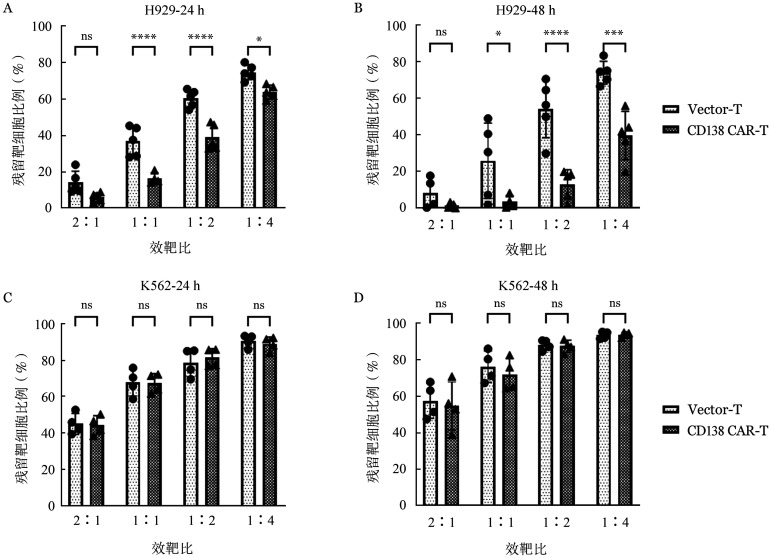
流式细胞术检测Vector-T和CD138嵌合抗原受体T细胞（CAR-T）与H929靶细胞共培养24 h（A）、48 h（B）及K562靶细胞24 h（C）、48 h（D）后残留靶细胞（实验重复5次，*P<0.05，***P<0.001，****P<0.0001，ns示差异无统计学意义）

脱颗粒试验提示，CD138（5G2）CAR-T细胞可以被CD138^+^靶细胞显著激活：与H929共培养4 h后，CD138（5G2）CAR-T细胞激活率为（25.78±3.35）％，显著高于Vector-T组细胞激活率（6.13±1.30）％（*P*<0.001）。而与CD138^−^靶细胞K562共培养后CD138（5G2）CAR-T及Vector-T细胞组均无明显激活［（6.06±1.13）％对（5.62±1.04）％，*P*＝0.940］（图7）。

**图7:**
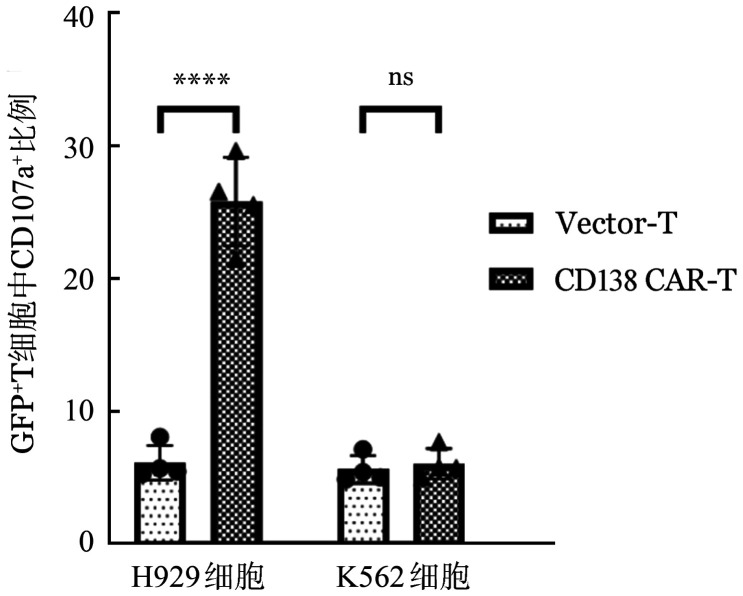
流式细胞术检测Vector-T及CD138嵌合抗原受体T细胞（CAR-T）与靶细胞共培养4 h CD107a^+^表达（实验重复4次，****P<0.0001，ns示差异无统计学意义）

免疫荧光法检测CD138（5G2）CAR-T细胞与MM细胞共培养48 h（效靶比为1∶1）上清中细胞因子分泌水平。结果显示：CD138（5G2）CAR-T与CD138^+^ H929细胞共培养后上清中IL-2、IFN-γ和TNF-α水平分别为（1 697.52±599.05）、（3 312.20±486.38）及（1 837.43±640.49）pg/ml，均显著高于Vector-T组［IL-2：（5.07±1.17）pg/ml，*P*<0.001；IFN-γ：（9.28±1.46）pg/ml，*P*<0.001；TNF-α：（8.75±1.65）pg/ml，*P*<0.001］。而与CD138^−^ K562细胞共培养后CD138（5G2）CAR-T细胞分泌的细胞因子较Vector-T组无显著升高［IL-2：（13.52±2.42）pg/ml对（13.69±2.61）pg/ml，*P*＝1.000；IFN-γ：（6.42±0.67）pg/ml对（7.02±0.52）pg/ml，*P*＝1.000；TNF-α：（9.28±1.46）pg/ml对（6.38±1.09）pg/ml，*P*＝1.000］（图8）。

**图8:**
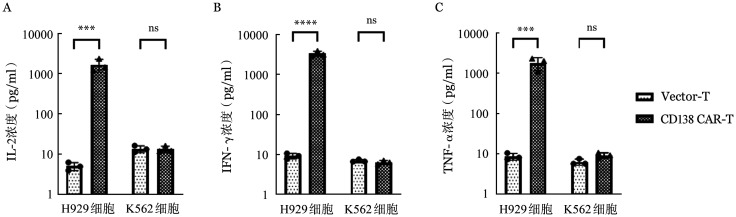
免疫荧光法检测Vector-T和CD138嵌合抗原受体T细胞（CAR-T）和靶细胞共培养48 h培养上清细胞细胞因子IL-2（A）、IFN-γ（B）和TNF-α（C）释放水平（实验重复3次，****P<0.0001，***P<0.001，ns示差异无统计学意义）

## 讨论

CAR-T细胞疗法现已成为MM一种有效的治疗方式[6],[12]。全球已批准3种靶向B细胞成熟抗原（BCMA）CAR-T细胞治疗产品：idecabtagene vicleucel（ide-cel）、ciltacabtagene autoleucel（cilta-cel）和CT103A，用于复发难治MM（R/R MM）的治疗[13]–[15]，并已展现出较好的治疗反应。然而接受BCMA CAR-T治疗的患者可能会面临因BCMA表达低或缺失而导致的肿瘤复发[12]，或先前接受过BCMA靶向治疗患者BCMA CAR-T细胞治疗反应率较差[16]–[17]等一系列问题。且MM在疾病进展中常出现多个亚克隆，遗传和表型异质性较高[18]，因此需要开发其他MM治疗的理想靶点。

CD138高表达于恶性浆细胞表面，且参与细胞增殖、血管生成、侵袭和转移等肿瘤发生发展过程。前期CD138 CAR-T临床试验展现出可接受的安全性及有效性[19]–[20]，提示CD138可以成为MM CAR-T细胞治疗的理想靶点。

CAR结构中抗原识别区通过识别不同抗原表位及亲和力的差异，使CAR-T细胞产生不同的治疗效果[21]–[22]。MM是一种高度异质性的疾病，全新抗原识别区序列的发现可能为MM细胞治疗带来新的可能；与此同时，不同的抗原识别表位，可能为MM肿瘤细胞表面抗原表位突变而介导的CAR-T治疗后肿瘤复发问题提供新的解决策略。本研究中我们制备并筛选出一株全新CD138单克隆抗体，并获得其scFv序列用于CD138 CAR的构建，希望通过不同的抗原识别表位为MM的治疗提供新的可能。

本次研究结果表明，5G2杂交瘤株产生的单克隆抗体可以特异性识别细胞表面的CD138抗原，且亲和力高。以5G2可变区序列为抗原识别区构建的CD138（5G2）CAR，可以成功表达于T细胞表面，并有效结合CD138蛋白。细胞表型检测发现，与Vector-T组相比，CD138（5G2）CAR的表达可以促进T细胞向T_CM_及T_SCM_方向分化，而减少终末分化T细胞的比例，有利于T细胞更持久的作用于肿瘤细胞。细胞毒性实验提示，CD138（5G2）CAR-T细胞可以被CD138^+^靶细胞特异性激活，脱颗粒水平增加，并能够有效发挥细胞毒作用，杀伤CD138^+^肿瘤细胞。而对CD138^−^靶细胞无明显脱靶效应。因CD138分子在细胞黏附、细胞-基质相互作用及细胞-细胞相互作用中发挥重要作用，虽然靶向CD138治疗可以有效减少肿瘤负荷，可能存在引起肿瘤播散的潜在风险。因此CD138 CAR-T治疗与其他疗法的联合应用尤为重要，我们后续将会继续研究CD138 CAR-T在MM联合治疗中所发挥的作用。

综上所述，本研究成功制备、筛选得到5G2杂交瘤株，可稳定分泌特异性识别CD138的单克隆抗体，以其可变区序列构建的CD138（5G2）CAR-T细胞可以有效杀伤CD138^+^骨髓瘤细胞，并对CD138^−^靶细胞无明显脱靶效应，为CD138抗原检测及MM的治疗提供了新的选择。
